# Incidence of melancholic depression by age of onset and gender in the Lundby population, 1947–1997

**DOI:** 10.1007/s00406-022-01506-5

**Published:** 2022-11-05

**Authors:** Linnéa Nöbbelin, Mats Bogren, Cecilia Mattisson, Louise Brådvik

**Affiliations:** Department of Clinical Sciences, Division of Psychiatry, Lund University, S-221 85 Lund, Sweden

**Keywords:** Melancholia, Depression, Incidence, Psychotic depression, Epidemiology

## Abstract

Whether melancholic depression is a distinct syndrome or not has long been debated. There are few studies providing information about the epidemiology of melancholic depression. In this study, we investigate the incidence rates, overall as well as by gender and age of onset of melancholic depression according to Taylor and Fink and corresponding DSM–IV disorders: major depressive disorder (MDD) with melancholic specifier, MDD with psychotic features, MDD with postpartum debut and bipolar depression in the Lundby population. Incidence rates with 95% confidence intervals were calculated. The incidence rate of melancholic depression was 0.48 (CI 0.36–0.61) per 1000 person-years under risk. The rates of the corresponding DSM-IV disorders were as follows: MDD with melancholic specifier 0.38 (CI 0.27–0.49), MDD with psychotic features 0.13 (CI 0.07–0.21), MDD with postpartum debut 0.02 (CI 0.00–0.06) and bipolar depression 0.04 (CI 0.01–0.10). Females had a significantly higher incidence rate, with a peak in age group 40–49, in melancholic depression according to Taylor and Fink and MDD with melancholic specifier. There was no gender difference in incidence rates of MDD with psychotic features or bipolar depression. The diagnoses were set in retrospect and the number of subjects with MDD with postpartum debut and bipolar depression was low. Incidence of melancholia was low in the Lundby Study. There was a female preponderance to become melancholically depressed in line with research on undifferentiated depression.

## Introduction

Depression is a common disorder estimated to globally affect 322 million people [1]. The DSM-diagnosis major depressive disorder (MDD) [2] has been criticized for being too heterogeneous including states that have nonspecific depressive features in common [3, 4]. It has been debated whether depression is constituted of qualitatively different pathophysiological syndromes or if different depressive presentations merely differ quantitatively. Melancholic depression has attracted specific interest [5]. For a long time, a binary view, dividing depression into endogenous/melancholic and non-endogenous reactive/neurotic forms, dominated, but in the 20th century, the binary view was challenged by a unitary view arguing that depressive states only differ on a dimensional scale and melancholic depression came to be viewed as a severe form of depression rather than a specific syndrome [5]. Lately however, the debate on whether melancholic depression should be resurrected as a diagnosis distinct from other types of depression has been rejuvenated [4, 5]. In some studies, melancholic depression has been shown to have a poorer response to hospitalization, placebo and psychotherapy [6] and a better response to electroconvulsive therapy (ECT) and tricyclic antidepressants (TCA) [7] than non-melancholic depression.

Throughout the 20th and 21th century, several diagnostic constructs of melancholic depression apart from the melancholic specifiers in DSM-III [8], DSM-IIIR [9], DSM-IV [2] and DSM-5 [10] have been developed to define a melancholic diagnostic group separated from other types of depression [11], e.g., the Core system [12], the Sydney Melancholia Prototype Index (the SMPI) [13] and Taylor and Finks concept of melancholia [14]. Most of the constructs, especially the DSM-melancholic specifiers, are based on the presence of a specific number of criteria, mainly symptom criteria. Some symptom criteria are more prevalent in the constructs, e.g., psychomotor disturbances, anhedonia, early awakening and diurnal variation. One system, the Core system, is based solely on the measurement of different aspects of psychomotor disturbance [12]. However, some constructs include non-symptom criteria such as absence of premorbid personality deviance in DSM-IIIR, and no adequate psychogenesis and average amount of childhood stressful life-events in the SMPI. Taylor and Fink [15] approach the subject from a different perspective primarily arguing that depressive syndromes should be divided into melancholic and non-melancholic syndromes as opposed to the current division between unipolar and bipolar syndromes. They argue that MDD with melancholic, psychotic or catatonic features, bipolar depression, puerperal depression, and abnormal bereavement are essentially melancholic syndromes with similar features, i.e., presence of psychomotor disturbances, anhedonia and vegetative signs, and comparable illness course, response to treatment and dexamethasone suppression test results [14, 15]. The use of different diagnostic constructs on a population does not yield completely overlapping patient groups [11, 16] making comparisons between studies using different constructs difficult.

There are very few epidemiological studies in the general population providing information about the prevalence and incidence of melancholic depression and other subtypes of depression, e.g., atypical depression and anxious depression [17]. Melancholic depression and depression with psychotic features have mostly been studied in enriched samples such as inpatients or severe psychiatric outpatients samples [18]. However, there is some evidence of melancholic depression being treated frequently also in less specialized settings such as primary care [19].

The cumulative incidence of melancholic depression according to DSM–IV and atypical depression combined in the Zurich study was 4.1%, whereas for melancholic depression only during the follow-up it was 7.1 [20]. In this study, a sample of 592 young adults were followed for 21 years and data were collected through six waves of interview. The attrition rate was high, 38.5%. The cumulative incidence of melancholic and atypical depression combined was calculated from the number of patients who had been diagnosed with both melancholic and atypical depression in separate episodes. In a community sample in northeast Ireland, Baldwin et al. [21] found an incidence rate of DSM-IV MDD with psychotic features of 6.4 annually per 100 000 persons. In the Netherlands mental health survey and incidence study-2 (NEMESIS-2), the incidence rate of DSM-IV bipolar depression was 0.13 per 100 person-years under risk [22]. To our knowledge, there are no population-based studies on incidence or prevalence of melancholic depression using Taylor and Finks concept of melancholia.

The Lundby Study is a prospective study of a complete population making it especially suitable for studies of incidence. Previously, a study on gender differences in the first incidence by age at onset of subtypes of depression, including melancholic depression according to the division of DSM-IV depressive syndromes into melancholic and non-melancholic depression proposed by Taylor and Fink, has been published [23]. In this study, only first episodes of depression with severe or very severe impairment were evaluated melancholic or non-melancholic. Results from the previous study showed no gender difference in the first incidence of melancholic depression according to Taylor and Fink and depression with severe impairment defined by the Lundby diagnostic system, respectively, as opposed to most other subgroups of depression, including DMS IV major depressive disorder. To find more episodes of melancholic depression to underpin incidence calculations, in the current study, we go on to investigate the entire course of depression, including recurrent onsets as well as episodes of medium impairment. We have also calculated the incidence rates of corresponding DSM-IV diagnoses.

The aim of this study is to investigate the incidence rates by age of onset and gender of melancholic depression according to Taylor and Fink and of the corresponding DSM-IV disorders (i) MDD with melancholic specifier, (ii) MDD with catatonic specifier, (iii) MDD with psychotic features (iv) MDD with postpartum debut and (v) bipolar depression.

## Methods

### The Lundby Study

The Lundby Study follows two overlapping cohorts of 3563 subjects (1823 men and 1740 women). All who resided in two adjoining parishes in the south of Sweden, either in 1947 or 1957, were included. Four field investigations have taken place: 1947, 1957, 1972 and 1997. The attrition rate was low, between 1 and 2% in the follow-ups in 1957 and 1972, and 6% in 1997. Psychiatrists conducted all investigations. Information was gathered through semi-structured interviews, key informants, registers and patient records. The field investigations and the study population have been described elsewhere [24].

### Diagnostic assessment

When the Lundby Study began, the DSM system was not in use and consequently has only been applied in the 1997 investigation. Throughout the entire study, the Lundby diagnostic system was used. The psychiatric diagnoses were set in agreement by the team of psychiatrists using all available information from interviews, key informants, registers and case notes. The degree of impairment for every episode of psychiatric disorder was rated as very severe, severe, medium, or mild [25]. In 1997, the degrees of impairment were approximated to Global Assessment of Functioning (GAF)-scores [2]: mild degree of impairment corresponds to GAF 61–70, medium degree corresponds to GAF 51–60, and severe and very severe to GAF 1–50 [26]. In 1997, in addition to Lundby diagnoses, diagnoses according to DSM-IV [2] including GAF-scores, and the ICD-10 [27] were recorded.

The criteria for depression in the Lundby diagnostic system are as follows:

“Lowered mood, depressive feelings, tendency to guilt feelings, gloomy outlook, reduced activity, lack of initiative, reduced self-esteem, lowered enjoyment of life and a feeling of low vitality, anxiety and fear. Has more difficulty than usual, and is often unable to carry out his daily responsibilities. Sometimes retardation is present. The subject is often worse in the morning and better towards the evening. Often, he has sleep disturbances and wakes up in the early morning. Loss of appetite and weight” [28].

The Lundby diagnostic system also includes the diagnosis depression + , which includes cases with predominant depressive symptoms although accompanied by other mental symptoms, e.g., anxiety and obsessive symptoms. Sixty percent of the episodes with Lundby depression of medium or worse impairment 1947–1997 corresponded to DSM-IV MDD, whereas the rest could be classified as other DSM-IV subtypes of depression or adjustment disorder with depressed mood [29]. Lundby depression of mild impairment does not reach the threshold for DSM-IV caseness [30].

Melancholic depression was diagnosed in retrospect in accordance with Taylor and Finks division of depressive disorders into melancholic and non-melancholic syndromes [15]. Melancholic depression according to Taylor and Fink is a conglomerate of several DSM-IV syndromes. Taylor and Fink argue that MDD with melancholic, psychotic or catatonic features, bipolar depression, puerperal depression, and abnormal bereavement are melancholic syndrome, whereas depressive syndromes such as MDD without specifier, MDD with atypical specifier and MDD with seasonal pattern, dysthymia, brief or minor depression and adjustment disorder with depressed mood are non-melancholic disorders. The Lundby data were translated into Taylor and Fink’s division of depressive disorders via a reassessment of the Lundby data according to DSM-IV. Depression episodes of medium to very severe impairment according to the Lundby diagnostic system were reassessed using all available information: interview protocols, patient records and extracts from data records gathered during previous field investigations, in accordance with DSM-IV diagnostic constructs of: (i) MDD with melancholic specifier, (ii) MDD with catatonic specifier, (iii) MDD with psychotic features (iv) MDD with postpartum debut and (v) bipolar depression [31]. All first time episodes, whether the first time ever or recurrent episode of depression, of any of the abovementioned DSM-IV syndromes were added in the calculations of the incidence rate of melancholic depression according to Taylor and Fink*.*

Taylor and Fink [14] have also proposed a classic item based diagnostic construct of melancholia including the items anhedonia, psychomotor disturbances, vegetative signs and specific laboratory test results. Using this construct, all items should be present to deem a depressive episode as melancholic. However, this diagnostic construct of melancholic disorder has not been used in the current study due to the lack of laboratory data.

### Statistical analysis

Incidence rates with 95% confidence intervals for melancholic depression according to Taylor and Fink and corresponding DSM-IV disorders were calculated. The calculations were based on the study subjects free from any type of depression, dementia or psychosis at inclusion. Incidence rates were calculated by dividing the number of cases by person-years under risk of the disorder. Subjects were considered to be at risk of the disorder until falling ill in the disorder, dying or study termination. When calculating the person-years under risk of developing depression the time since onset of organic syndrome, age psychosis, schizophrenia and other types of psychosis according to the Lundby diagnostic system in afflicted subjects were subtracted. The rationale behind this was that in the Lundby diagnostic system, these diagnoses were of higher order and excluded the possibility of being diagnosed with diagnoses of lower order, including depression. Out of the 3563 subjects 77 were excluded due to having had a depressive episode before inclusion and 64 due to being diagnosed with higher order Lundby diagnoses before inclusion and never recovering.

## Results

### Risk sampl*e*

Demographic data on the 3420 subjects included in the calculations divided into groups based on whether having had melancholic depression according to Taylor and Fink, other types of depression or no type of depression during the follow-up is shown in Table 1. The median time of follow-up was 480 months (IQR 263 months, range 1–600 months).

**Table 1:** Demographic data on subjects in the Lundby cohort at risk of developing depression (*N* = 3420) divided into groups based on depressive illness during the follow-up at study start

Variable	Non-cases (*N* = 2988 (87.4%))	Other depression-cases (*N* = 373 (10.7%))	Melancholia-cases^a^ (*N* = 59 (1.7%))
Gender			
Women	1394 (46.7%)	227 (61.0%)	40 (68.0%)
Men	1594 (53.3%)	146 (39.0%)	19 (32.0%)
Mean age^b^	32.2 (22.45)^c^	24.1 (16.5)^c^	30.6 (16.0)^c^
Socioeconomic status^d^			
Blue collar^e^	1672 (56.0%)	215 (58.6%)	38 (64.4%)
White collar^f^	833 (27.9%)	117 (31.4%)	10 (16.9%)
Self-employed^g^	468 (15.7%)	40 (10.7%)	11 (18.6%)
No information	15 (0.5%)	1 (0.3%)	0 (0.0%)
Marital status^d^			
Never married	410 (13.7%)	34 (9.1%)	5 (8.5%)
Married/co-habiting	1939 (64.9%)	226 (60.6%)	33 (55.9%)
Widowed	459 (15.4%)	65 (17.4%)	10 (16.9%)
Divorced/separated	178 (6.0%)	48 (12.9%)	11 (18.6%)
No information	2 (0.1%)	0 (0.0%)	0 (0.0%)

^a^Melancholic depression according to the division of DSM-IV depressive syndromes into melancholic and non-melancholic depression proposed by Taylor and Fink [10]

^b^Mean age at inclusion in the Lundby study

^c^Standard deviation

^d^Latest known status

^e^Unskilled, semiskilled, and skilled manual workers

^f^Assistant and intermediate non-manual employees, employed and self-employed professionals, higher civil servants and executives

^g^Other than professionals

### Incident cases

During the follow-up, 59 subjects (1.7%) had at least one episode of melancholic depression defined in accordance with Taylor and Fink. In these subjects, the first episode of melancholic depression was not always the first ever episode of depression as previous episodes could be non-melancholic. Three hundred seventy-three subjects (10.7%) had only non-melancholic types of depression. In the group with at least one episode of melancholic depression, the mean age at onset of the first depressive episode was 47.0 years (SD 17.2), and in the group with other types of depression, the mean age at first onset was 46.0 years (SD 15.0). Among the subjects afflicted by melancholic depression, the mean age of onset of the first melancholic depressive episode was 49.2 years (SD 15.0 years). In subjects with melancholic depression, the first episode of depression was of severe or worse impairment in 45.8% of the cases, and in subjects with non-melancholic depression, the first episode was severe or worse in 18.2%.

Among the 59 subjects with melancholic depression, during the follow-up, 39 had more than one depressive episode. However, out of the 39, only 9 (23.1%) were melancholic at every episode and only 17 (43.5%) more than once. Nevertheless, in the 39 with repeated episodes, the median episode at which melancholic depression was first diagnosed was the first (range 1–18, IQR 1). And looking at all the 59 subjects with melancholic depression at least once 44 had melancholic depression as their first depressive episode, nine as their second and six later.

**Fig. 1:**
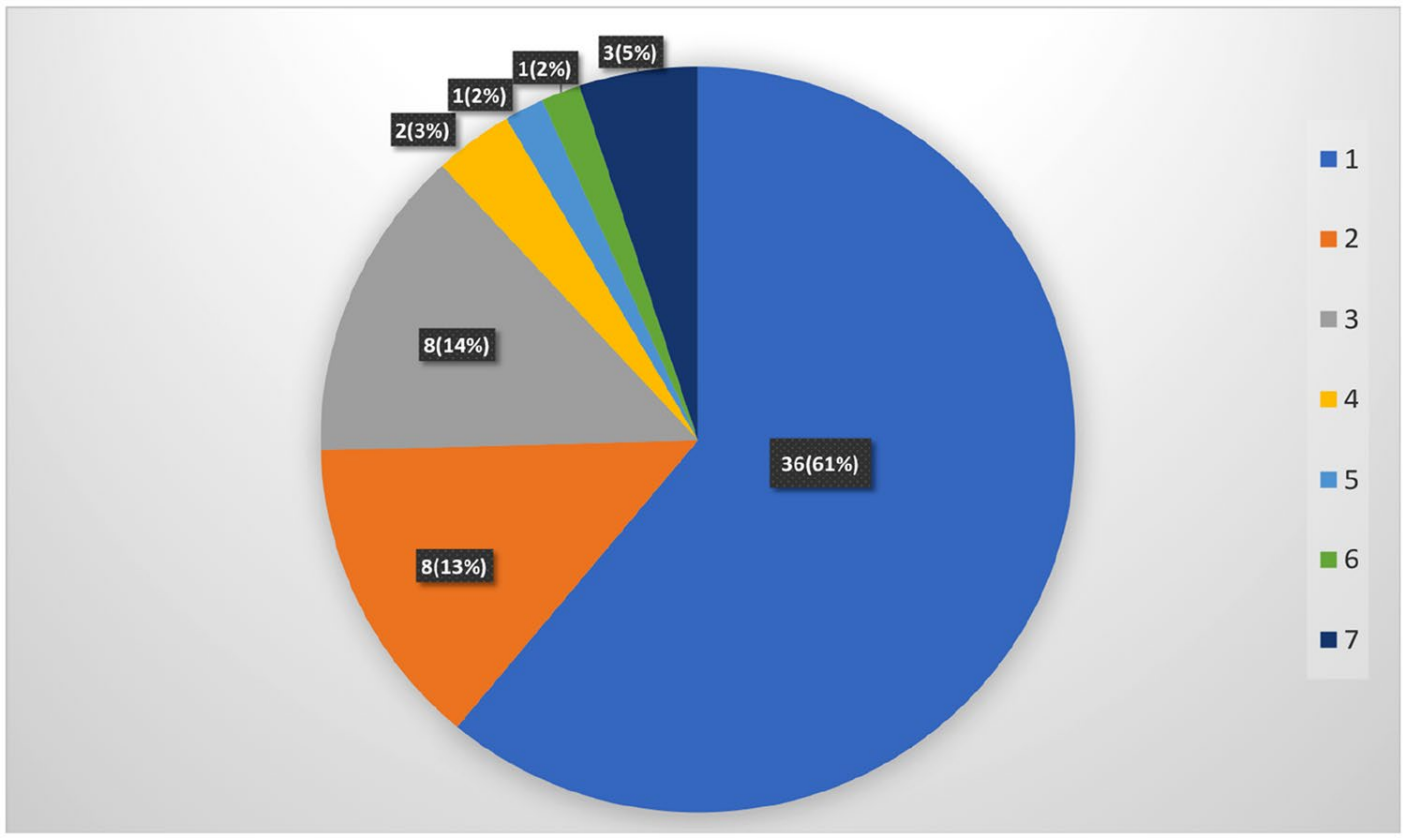
Subjects afflicted by melancholic depression (*N* = 59), according to Taylor and Fink (2006), in the Lundby cohort divided into groupings based on corresponding DSM-IV disorders/specifiers [2] present during the course of illness. ^1^MDD with melancholic specifier. ^2^MDD with psychotic features. ^3^MDD with melancholic specifier and psychotic features. ^4^MDD with melancholic specifier and postpartum debut. ^5^Bipolar depression and depressive episodes with melancholic specifier. ^6^Bipolar depression and depressive episodes with melancholic specifier and psychotic features. ^7^Bipolar depression and depressive episodes with psychotic features

During both within an episode of melancholic depression as well as during the course of a recurring depressive illness with melancholic depressive episodes according to Taylor and Fink, it was possible for the same subject to be diagnosed with more than one corresponding DSM–IV depressive disorder/specifier. Among the 59 subjects with melancholic depression according to Taylor and Fink, 50 had at least one depressive episode with melancholic specifier, 23 a depressive episode with psychotic features, two a depressive episode with postpartum onset and five were diagnosed with bipolar depression according to DSM-IV. No depressive episodes with catatonic features were found. The different groupings based on the different DSM-IV depressive disorder/specifier combinations in subjects with melancholic depression according to Taylor and Fink is illustrated in Fig. 1. In the five subjects with DSM-IV bipolar depression, all had depressive episodes with either melancholic specifier or psychotic features or both during their course of illness. The two cases with postpartum depression both had DSM-IV MDD with melancholic specifier.

### Overall incidence proportions and rates

Table 2 shows the incidence proportions and rates of melancholic depression and the corresponding DSM-IV disorders. It also shows the proportions of the total depressed subpopulation of the sample with melancholic depression and the parallel DSM-disorders.

**Table 2:** Overall incidence rates of melancholic depression, according to Taylor and Fink, and corresponding DSM-IV diagnoses in risk sample (*N* = 3486)

	*N*	Incidence proportion	Incidence rate^b^ (95% CI)^c^	Percentage of depressed population^a^
Melancholic depression^d^	59	1.7%	0.48 (0.36–0.61)	14.0%
DSM-IV disorders^e^				
MDD with melancholic specifier	46	1.3%	0.38 (0.27–0.49)	10.6%
MDD with psychotic features	16	0.5%	0.13 (0.07–0.21)	3.7%
MDD with postpartum debut	2	< 0.1%	0.02 (0.00–0.06)	< 0.1%
Bipolar depression	5	0.1%	0.04 (0.01–0.10)	1.2%

^a^*N* = 432

^b^Incidence rate per 1000 person-years under risk

^c^95% confidence interval

^d^According to the division of DSM.IV depressive syndromes into melancholic and non-melancholic depression proposed by Taylor and Fink [10]

^e^DSM-IV [2]

### Incidence rates by gender

Incidence rates in males and females and female/male incidence rate ratios of melancholic depression and corresponding DSM-IV diagnoses are shown in Table 3. The incidence rates of melancholic depression and MDD with melancholic specifier were significantly higher among females. There was no significant difference in incidence rates between the genders when looking at MDD with psychotic features and bipolar disorder.

**Table 3:** Gender-specific incidence rates and female/male incidence rate ratios of melancholic depression, according to Taylor and Fink, and corresponding DSM-IV disorders in the Lundby cohort

	Incidence rate^a^ (95% CI)		Incidence rate ratio (95% CI)	*P* value
	Females	Males		
Melancholic depression^b^	0.66 (0.46–0.87)	0.31 (0.19–0.48)	2.13 (1.21–3.91)	< 0.01^✻^
DSM-IV disorders^c^				
MDD with melancholic specifier	0.55 (0.36–0.74)	0.20 (0.11–0.36)	2.79 (1.41–5.94)	< 0.01^✻^
MDD with psychotic features	0.15 (0.07–0.28)	0.11 (0.05–0.23)	1.31 (0.43–4.13)	0.78
Bipolar depression	0.03 (0.00–0.12)	0.05 (0.01–0.14)	0.68 (0.06–5.91)	1.00

^a^Incidence rate per 1000 person-years under risk

^b^According to the division of DSM-IV depressive syndromes into melancholic and non-melancholic proposed by Taylor and Fink [10]

^c^[2]

^✻^Statistically significant

### Incidence rates by age at onset and gender

**Fig. 2:**
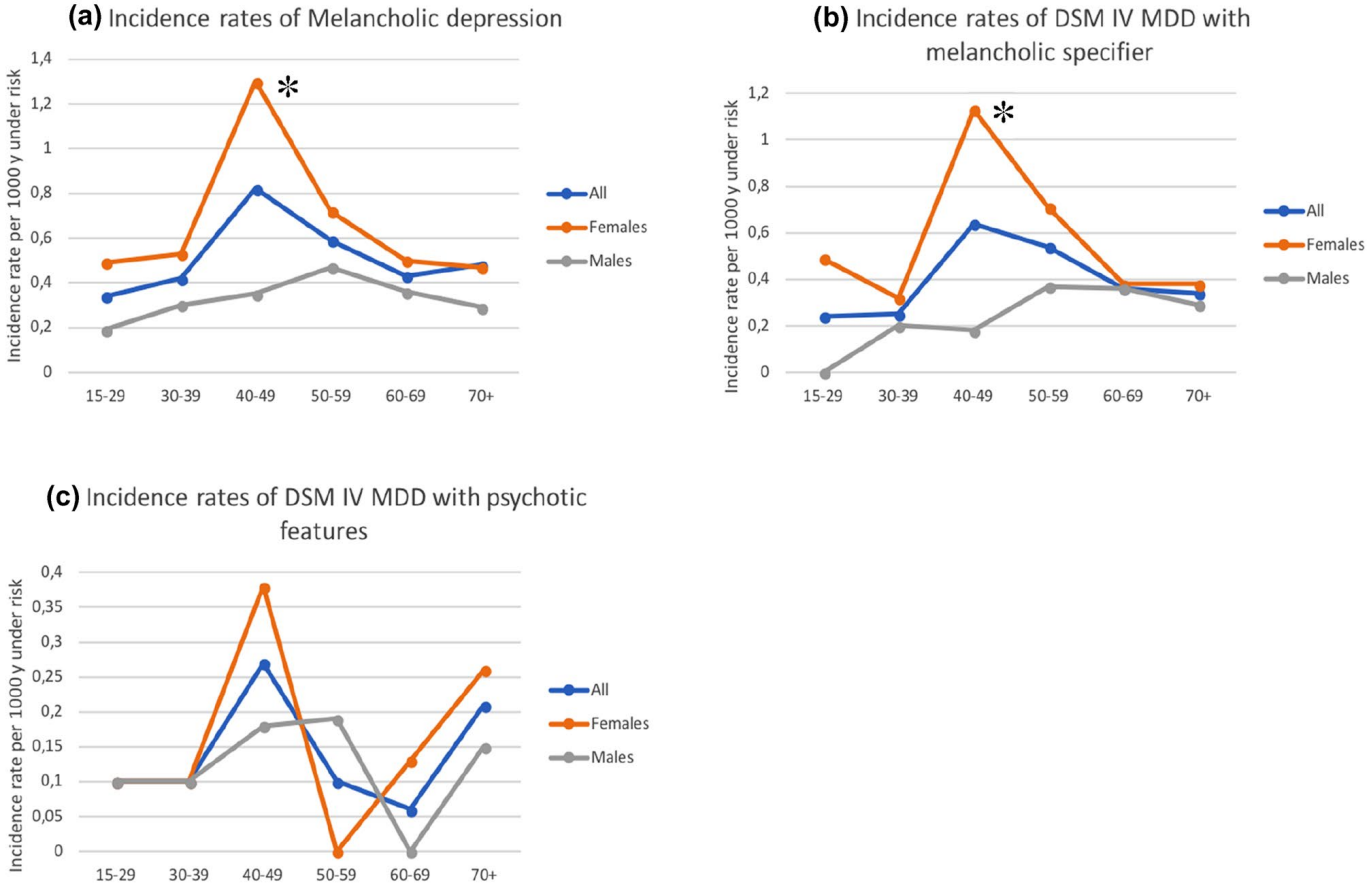
Incidence rates of melancholic depression by age at onset and gender grouped into: **a** melancholic depression according to the division of DSM-IV depressive syndromes into melancholic and non-melancholic depression proposed by Taylor and Fink [10], **b** DSM-IV MDD with melancholic specifier and **c** DSM-IV MDD with psychotic features [2]. Incidence rate per 1000 person-years under risk. ^✻^statistically significant difference between incidence rates of females and males

Incidence rates of melancholic depression, MDD with melancholic specifier and MDD with psychotic features by age at onset and gender are shown in Fig. 2. Rates for bipolar depression and MDD with postpartum debut were not possible to calculate due to the small number of cases. In all diagnostic groups, the highest overall incidence rate was in the age group 40–49. Females had a higher incidence rate of both melancholic depression and MDD with melancholic specifier compared to males in almost all age groups but the gender difference was only significant in age group 40–49. The incidence rate of MDD with psychotic features fluctuated greatly in the different age groups due to a small number of cases and no gender difference could be shown.

## Discussion

### Strengths and limitations

The strength of this study is its uniqueness in prospectively studying mental disorders in a non-selected community-based setting using data gathered from semi-structured interviews conducted by psychiatrists. The low attrition rate as well as the setting minimizes selection bias. The long follow-up time, 50 years, as well as the gathering of information from several sources; interviews, key informants, case records and registers, ensures a good case-finding rate.

However, there are a few limitations. The small size of the study population meant that there were too few cases of DSM-IV bipolar disorder and especially depression with postpartum debut to reliably estimate incidence rates and there were no cases of MDD with catatonic specifier. Recall bias is a limitation and probably biased the incidence rates downward, although recall bias was to some extent compensated by the external sources of information. Subjects ill at inception were not included, which also might have biased the incidence rates downward. The Lundby Study covers a period in which different diagnostic paradigms dominated making retrospective reevaluation necessary, which might have impacted the pattern of diagnoses. When looking at the incidence of melancholic depression, the retrospective evaluation has most probably biased the incidence rates downward due to lack of data in some threshold cases.

It is important to note that Taylor and Fink in a review [14] suggested a diagnostic construct of melancholic depression with the following criteria: anhedonia, psychomotor disturbances, vegetative signs and specific laboratory test results, which all should be present to deem a depression melancholic. This diagnostic construct has not been used due to lack of data on laboratory test results in the Lundby Study and the group melancholic depression according to Taylor and Fink is a conglomerate DSM-IV disorders deemed melancholic by Taylor and Fink [15].

### Main findings

The main aim of this study was to investigate the overall as well as gender- and age-specific incidence rates of melancholic depression according to Taylor and Fink and of corresponding DSM-IV diagnoses: MDD with melancholic specifier, MDD with psychotic features, MDD with postpartum onset and bipolar depression. The results showed a significantly higher overall incidence rate among females of melancholic depression and MDD with melancholic specifier; however, when looking at specific rates, the gender difference was only significant in age group 40–49 where females had a distinct incidence peak. There was no gender difference in incidence rates of MDD with psychotic features and bipolar depression.

### Incidence rates

The cumulative incidences in the Zurich Study of melancholic depression (DSM-IV melancholic specifier)—4.1% for combined melancholic and atypical depression and 7.1% for melancholic depression only—are neither directly comparable to the incidence rate nor the incidence proportion found in the Lundby Study, 1.3%. Nevertheless, as the follow-up time in the Lundby Study was longer, one would suspect a higher incidence proportion of melancholic depression in the Lundby Study when looking at the cumulative incidence found in the Zurich Study. Reasons for the low incidence proportion might be the older population in the Lundby Study, underreporting due to recall bias and the retrospective diagnosis of melancholic depression. During the 21 years’ follow-up in the Angst study [20], six waves of interview were conducted whereas only four field investigations in fifty years have been conducted in the Lundby Study. When diagnosing retrospectively, the inability to access further information might lead to a pattern of exclusion in threshold cases. The high attrition rate in the Zurich Study [20] may influence the results from that study.

When studying the incidence of psychotic depression, all previous studies, to our knowledge, have used data from inpatients and outpatients and estimated the frequency in a corresponding community sample. Baldwin et al. [21] found an incidence rate of DSM-IV MDD with psychotic features of 6.4 annually per 100000 persons in a community sample in northeast Ireland. Our study found a higher rate, 0.13 (CI 0.07–0.21) per 1000 person-years under risk and if converted, 13 annually per 100000 persons. One reason for this might be the different study settings. Some of the subjects suffering from psychotic depression in the Lundby cohort did not seek healthcare at the time of their depression but the diagnosis could be given based on interviews with the subject and relatives describing the episode. There might be some cases lost when focusing on inpatients and outpatients only. In a review of psychotic depression [32], a meta-analysis showed that the proportion of psychotically depressed among all depressive patients was 28% using data from both studies with in- and outpatients. However, it was 19% if only data from studies on outpatients were used. In our study, the corresponding percentage was 3.7%, which is significantly lower and most readily explained by the different settings, patients as compared to a population sample.

In a community study, the NEMESIS-2, the incidence rate of DSM-IV bipolar depression was 0.13 per 100 person-years under risk [22], which is distinctly higher than the incidence in our study, 0.04 (CI 0.01–0.10) per 1000 person-years under risk. In the NEMESIS-2, information was gathered by lay-men using the CIDI [33] and the study sample was chosen using a sampling procedure from the whole population of the Netherlands. In the Lundby Study, the diagnoses were set by a team of psychiatrists using information from several sources, including semi-structured interviews conducted by psychiatrists and the study sample was a geographically defined entire population. The period between interviews was 3 years in the NEMESIS-2 [22] and 10–25 in the Lundby Study. The difference in methods might in part explain the lower incidence estimate in the Lundby Study. Notwithstanding that the cases of bipolar depression in the current study are rather low and should be interpreted with caution. The two cases of MDD with postpartum onset were too few to reliably estimate an incidence rate.

### Incidence rate by gender and age of onset

A previous study [23] on gender differences in incidence rates of subtypes of depression in the Lundby Study showed no difference in rates of severe melancholic depression according to Taylor and Fink and severe Lundby depression between genders. However, most other subtypes, non-melancholic depression, Lundby depression of medium impairment, DSM-IV mood disorder and DSM-IV major depressive disorder, had a higher female incidence rate and the gender gap was most evident in the age group 40–49. Contrasting to this earlier finding, in the current study, the pattern of incidence rates by gender and age of onset of melancholic depression and DSM-IV MDD with melancholic specifier is similar to the pattern of the incidence rates of non-melancholic depressive subtypes, including a female-male gender gap. The age- and gender-specific pattern in the current study is also consistent with other studies on undifferentiated depression showing that the gender gap in depression emerges early [34], increases with age [35] and is particularly noticeable in middle life [36]. The opposing findings in the previous and current studies on gender differences in depression in the Lundby Study seem contradictory as both are based on the same material. However, in the previous study, only first depressive episodes of severe or very severe impairment were evaluated as melancholic or non-melancholic, whereas in the current study all episodes of depression of medium impairment or more were evaluated irrespective of when they occurred in the course of a recurrent illness. Thus, the change of gender distribution might be due to inclusion of episodes of medium impairment or of later episodes.

Post et al. [37] have suggested that each episode in a recurrent depressive illness changes psychopathology through a sensitization process resulting in longer, more severe (more often psychotic), more frequent episodes and self-acting course over time. Subjects with melancholic depression at first ever depressive episode and subjects with melancholic depression diagnosed first at later episodes might as consequence constitute different diagnostic groups, with the later diagnostic group reflecting the characteristics of a group with recurrent depressive illness in general, with changing phenotype over time, rather than a group with a distinct melancholic depressive type. Risk factors for melancholic depression at later episodes in a recurrent depressive disorder might also be different from risk factors for melancholic depressive illness at first ever depressive episode and as a result the group characteristics might change when including both in the analysis of incidence. However, most subjects, 74.6%, included in the current study had their first episode of melancholic depression as the first ever depressive episode minimizing a pattern of melancholic traits developing during a recurrent course and an effect of secondary etiological factors. Therefore, the differing results between the previous and current study are better explained by the inclusion of melancholic depression with medium impairment in the current study. One artefactual factor that might explain the gender difference in the current study is a higher propensity among women than men to report and remember more symptoms when less severely depressed [38]. Such a gender difference in reporting would be less evident in more severe episodes making a diagnosis of melancholic depression more likely in women than men with depression of medium impairment, but more similar between sexes in depression of severe impairment. However, the non-existing gender difference in incidence and age-specific incidence in severe melancholic depression in the previous study could very well reflect the identical pattern in severe depression irrespective of presence of melancholic trait and may not necessarily suggest a distinct difference between melancholic and non-melancholic depression in gender- and age-specific patterns of incidence.

Studies on gender differences in the incidence or prevalence of melancholic depression have shown different results. One population study [39] showed a higher prevalence rate of DSM-IV MDD with melancholic specifier in females compared to males and in another study [20] on cumulative incidence of DSM-IV MDD with melancholic specifier the rate was higher in males compared to females. Studies on in-and/or outpatients show a male overrepresentation [19, 40] or gender neutrality [41] in the group with DSM-IV with melancholic specifier. The overrepresentation of males or gender neutrality in DSM-IV melancholic depression seems to be more consistent in specialized care-settings whereas results are more diverse in population-based settings. This might be a reflection of an equal risk of falling ill in severe depression between the genders [23] and the fairly consistent results of female preponderance in falling ill in depression in general [22] interacting with a bias toward a higher impairment of depression among in- and/or outpatients.

There were no gender differences in overall or age-specific incidence rates of DSM-IV MDD with psychotic features or bipolar depression This is consistent with the literature [22, 32], but should nonetheless be interpreted with caution due to the low number of cases.

### Melancholic depression as proposed by Taylor and Fink

The five subjects with bipolar disorder with onset during the study period had at least one depressive episode reaching the threshold for MDD with melancholic specifier and/or psychotic features during their course of illness supporting the idea [15] of similarity of phenotype between melancholic unipolar depression and bipolar depression. Contrary to this, there are some studies suggesting a connection between atypical depression and bipolar disorder, especially bipolar type II [42]. However, in the Lundby Study, all the subjects with bipolar disorder were best classified as bipolar type 1. This is probably a result of the features of bipolar type II being less severe and the diagnosis first being added in DSM-IV [31] leading to that the interviewers in earlier field studies failed to inquire after the symptoms. The gender difference in incidence rates in melancholic depression defined by the DSM specifier did not exist in either bipolar or psychotic depression, which would not support grouping these diagnoses together. However, the few cases of psychotic and bipolar depression limit interpretation.

## Conclusion

The incidence rates of and percentage of depressed individuals afflicted by melancholic depression or corresponding DSM-IV disorders were in general lower than in previous studies and especially studies based on in-and outpatients as expected. Melancholic depression according to Taylor and Fink’s division of DSM-IV depressive syndromes into melancholic and non-melancholic and MDD with melancholic specifier had a significantly higher female overall incidence rate with the gender difference being most distinct in the age group 40–49 where one can see an incidence peak among females. The pattern was similar to non-melancholic and DSM-IV major depressive disorder (without specifier) in the Lundby Study. However, MDD with psychotic features and bipolar depression had no gender difference in overall or age-specific incidence rates similar to severe depression in the Lundby Study.

## Data Availability

The data that support the findings of this study are available from the corresponding author, upon reasonable request. The data are not publicly available due to confidentiality.
